# Epidemiologic Features and Age-Related Differences in Management among Patients with Gastrointestinal Stromal Tumors in Japan: A National Cancer Registry Study

**DOI:** 10.1158/2767-9764.CRC-25-0074

**Published:** 2025-07-29

**Authors:** Hidekazu Hirano, Yoichi Naito, Toshirou Nishida, Takahiro Higashi, Tomoyuki Satake, Chigusa Morizane, Akira Kawai

**Affiliations:** 1 Department of Gastrointestinal Medical Oncology, National Cancer Center Hospital, Tokyo, Japan.; 2 Department of General Internal Medicine, National Cancer Center Hospital East, Chiba, Japan.; 3 Rare Cancer Center, National Cancer Center, Tokyo, Japan.; 4 Department of Surgery, Japan Community Healthcare Organization Osaka Hospital, Osaka, Japan.; 5 Division of Health Services Research, National Cancer Center, Tokyo, Japan.; 6 Department of Hepatobiliary and Pancreatic Oncology, National Cancer Center Hospital East, Chiba, Japan.; 7 Department of Hepatobiliary and Pancreatic Oncology, National Cancer Center Hospital, Tokyo, Japan.; 8 Department of Musculoskeletal Oncology and Rehabilitation, National Cancer Center Hospital, Tokyo, Japan.

## Abstract

Data on the epidemiology of gastrointestinal stromal tumor (GIST) and differences in its management according to age group are limited in Japan. We aimed to conduct an epidemiologic evaluation and describe age-related differences in management using data from Japan’s National Cancer Registry. We analyzed National Cancer Registry data of 21,426 patients with GIST between 2016 and 2019. We compared information on demographics, treatment, and prognosis across three age groups: pediatric and adolescent young adult (PAYA; ≤39 years), adult (40–74 years), and geriatric (≥75 years). Crude and age-adjusted annual incidences of GIST were 4.23 and 4.20 per 100,000 population, respectively. Regional variations in average age-adjusted annual incidence were observed among prefectures. The most common primary organs were stomach (72%), followed by the small intestine (21%). Geriatric patients represented 33% of the total population. Relative to PAYA and adult patients, geriatric patients were less likely to undergo surgery in the nonmetastatic setting (PAYA, 93%; adult, 93%; and geriatric, 87%; *P* < 0.001) or to receive chemotherapy in the metastatic setting (PAYA, 90%; adult, 87%; and geriatric, 61%; *P* < 0.001). Geriatric patients showed poorer 2-year overall survival relative to PAYA and adult patients in the nonmetastatic (PAYA, 98.5%; adult, 97.2%; and geriatric, 89.2%; *P* < 0.001) and metastatic (PAYA, 92.9%; adult, 79.2%; and geriatric, 54.7%; *P* < 0.001) settings. Geriatric patients comprised one third of the study population and were associated with less active treatment and an unfavorable prognosis.

**Significance:**

This is the first report presenting comprehensive Japanese epidemiologic data on GIST at a national level.

## Introduction

Gastrointestinal stromal tumors (GIST) are rare neoplasms arising from the gastrointestinal tract and are characterized by heterogeneity of clinicopathologic and molecular features. The annual incidence of GIST is approximately 1 to 2 per 100,000 population based on epidemiologic data outside Japan (1). Despite its rarity, several organizations have worked to develop guidelines for contributing to the standardization of treatment in clinical practice (2–4). Understanding the epidemiologic characteristics of GIST at the national level is essential for elucidating treatment practice and promoting clinical investigations.

GIST is primarily a disease of the older population. The median age at diagnosis is in the mid-60s (1). Older patients often have multiple comorbidities and a decline in their physical or cognitive function. Owing to the effects of aging, older patients with GIST have been underrepresented in pivotal clinical trials. Several reports are available to provide insights into clinical practice for older patients in real-world settings. Both surgery and adjuvant imatinib treatment for localized GIST are less likely to be performed in older patients (5). Older patients are reported to be more inclined to start imatinib treatment at reduced dosages relative to their non-older counterparts (6, 7). The clinical outcomes of older patients with GIST are comparable with those of non-older patients in the metastatic setting (5, 7–9). Nonetheless, the existing literature on older patients is scarce and is often based on studies with relatively small patient cohorts. Given the insufficient data, further exploration is warranted to better understand treatment patterns for older patients in clinical practice.

In Japan, the National Cancer Registry (NCR) was established in January 2016, consolidating data from all individuals diagnosed with cancer under a unified system. This novel framework has been devised to facilitate national cancer control measures by leveraging information on cancer incidence, survival rates, and treatment modalities. NCR data can be utilized as a reliable source for assessing information on various cancer types at a national level in Japan.

In this study, we investigated the epidemiologic characteristics of GIST in Japan and examined the differences in clinical characteristics and management between geriatric patients with GIST (age ≥75 years) and non-geriatric patients using NCR data. This study represents the first population-based epidemiologic investigation of GIST conducted at the national level in Japan.

## Materials and Methods

### Data source

We identified patients diagnosed with GIST who were registered in the NCR from January 1, 2016, to December 31, 2019, using a specific histologic code (International Classification of Diseases for Oncology, third edition, code: 8936). Information on population statistics was gathered from the Ministry of Health, Labour, and Welfare, Japan. Demographic variables, including the age at diagnosis, sex, primary site, extent of disease at diagnosis, detection mode, treatment patterns, and prognosis, were extracted. In the NCR, the category of “chemotherapy” includes molecular targeted agents such as tyrosine kinase inhibitors. In addition, the age-adjusted incidence rates of gastric cancer by prefecture from 2016 to 2019 were obtained from the NCR data provided by the Cancer Information Service, National Cancer Center, Japan (NCR, Ministry of Health, Labour, and Welfare).

### Statistical analysis

We calculated crude annual incidences as a number of new GIST cases during 2016 and 2019 divided by annual populations of 2016 to 2019. The incidences are reported per 100,000 population. Age-adjusted annual incidence was calculated using weighted proportions of the corresponding age groups according to the 2015 Japan Standard Population. Patients diagnosed with GIST at ages of ≥75 years, 40 to 74 years, and ≤39 years were classified into the geriatric, adult patients, and pediatric and young adult (PAYA) groups, respectively. We calculated the frequency (proportion) for categorical variables. The *χ*^2^ test was used to test the statistical difference in proportions. Pearson correlation coefficient was used to evaluate the correlation between variables. Overall survival (OS) was defined as the period from the date of the diagnosis to the date on which the patient was last contacted or died. OS was estimated using the Kaplan–Meier method, and comparisons were assessed using the log-rank test. Univariate analyses were conducted using a Cox proportional hazards model. Analyses of OS and treatment patterns were conducted after excluding patients with unknown clinical stage or autopsy as the mode of detection. *P* values of < 0.05 were considered to indicate statistical significance. All statistical analyses were performed using SPSS (ver. 28.0, IBM Co.) and R packages for Windows. In accordance with the guidelines of the NCR in Japan, absolute numbers for any subgroup with fewer than 10 individuals were not disclosed because of concerns about personal information protection.

### Data availability

The data generated in this study are available upon request from the corresponding author. The data are not publicly available because of privacy and ethical restrictions by law.

## Results

### Characteristics of study patients

**Figure 1:**
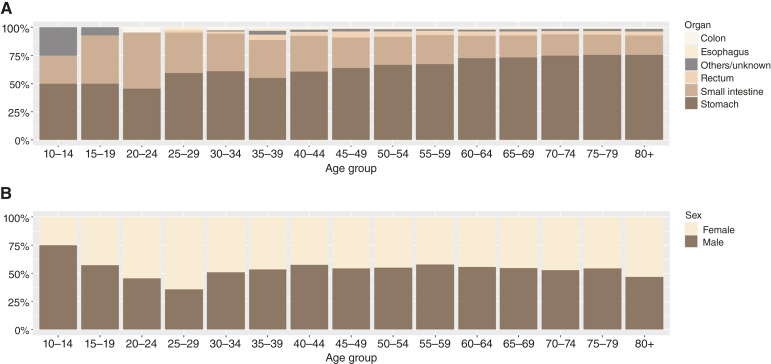
Distribution of patients according to the age group. **A,** By primary organ. **B,** By sex.

A total of 21,426 patients were registered in the NCR in Japan between 2016 and 2019 (Table 1). GIST occurred more frequently in men (53%) than in women (47%). The most common primary organ was the stomach (72%), followed by the small intestine (21%) and the rectum (4%). Most patients presented with localized disease (79%), whereas 5% of patients harbored distant metastasis at the time of diagnosis. The most common mode of detection was incidental diagnosis (46%), followed by symptoms (33%) and screening (16%). The 21,426 patients were classified into the PAYA (*n* = 441; 2%), adult (*n* = 13,898; 65%), and geriatric (*n* = 7,087; 33%) age groups. The proportion of each primary organ site varied according to age group (Fig. 1A). In all age groups, the most common primary organs were the stomach and small intestine. The incidence of stomach GIST increased with age, whereas the incidence of small intestine GIST decreased with age. Generally, there was a male predominance in most age categories; however, females were more prevalent in the 20 to 24 year, 25 to 29 year, and ≥80 year age groups (Fig. 1B). The PAYA group had a lower proportion of localized GIST (73%) than the adult (80%) and geriatric (77%) groups. Moreover, the PAYA group had a higher proportion of GIST detected because of symptoms (45%) than the adult (32%) and geriatric (35%) groups.

**Table 1:** Patient characteristics

Characteristic	Total (*N* = 21,426)		PAYA (*N* = 441; 2%)	Adult (*N* = 13,898; 65%)	Geriatric (*N* = 7,087; 33%)
Age, years	​	​	​	​	​
10–19	18	0%	4%	0%	0%
20–29	64	0%	15%	0%	0%
30–39	359	2%	81%	0%	0%
40–49	1,412	7%	0%	10%	0%
50–59	2,808	13%	0%	20%	0%
60–69	5,933	28%	0%	43%	0%
70–79	7,118	33%	0%	27%	48%
80+	3,714	17%	0%	0%	52%
Diagnosed year	​	​	​	​	​
2016	5,127	24%	23%	24%	24%
2017	5,474	26%	27%	26%	25%
2018	5,213	24%	26%	24%	25%
2019	5,612	26%	24%	26%	27%
Sex	​	​	​	​	​
Male	11,399	53%	51%	55%	50%
Female	10,026	47%	49%	45%	50%
Unknown	<10	0%	0%	0%	0%
Not reported	<10	0%	0%	0%	0%
Primary site	​	​	​	​	​
Esophagus	197	1%	2%	1%	1%
Stomach	15,499	72%	56%	71%	76%
Small intestine	4,410	21%	35%	22%	18%
Colon	160	1%	1%	1%	1%
Rectum	757	4%	3%	4%	3%
Other/unknown	403	2%	3%	2%	2%
Clinical stage	​	​	​	​	​
Localized	16,918	79%	73%	80%	77%
Regional lymph node metastasis	90	0%	2%	0%	1%
Invasion to adjacent structures	1,146	5%	7%	5%	5%
Distant	1,009	5%	5%	5%	5%
Unknown	2,263	11%	14%	9%	13%
Detection mode	​	​	​	​	​
Screening	3,499	16%	26%	21%	7%
Incidental diagnosis	9,922	46%	23%	44%	53%
Symptom	7,155	33%	45%	32%	35%
Autopsy	67	0%	0%	0%	1%
Unknown	783	4%	5%	3%	4%

Less than 10 cases is shown as “<10.” Because of rounding, the totals may not add up to exactly 100%.

### Epidemiologic features of GIST in Japan

**Figure 2:**
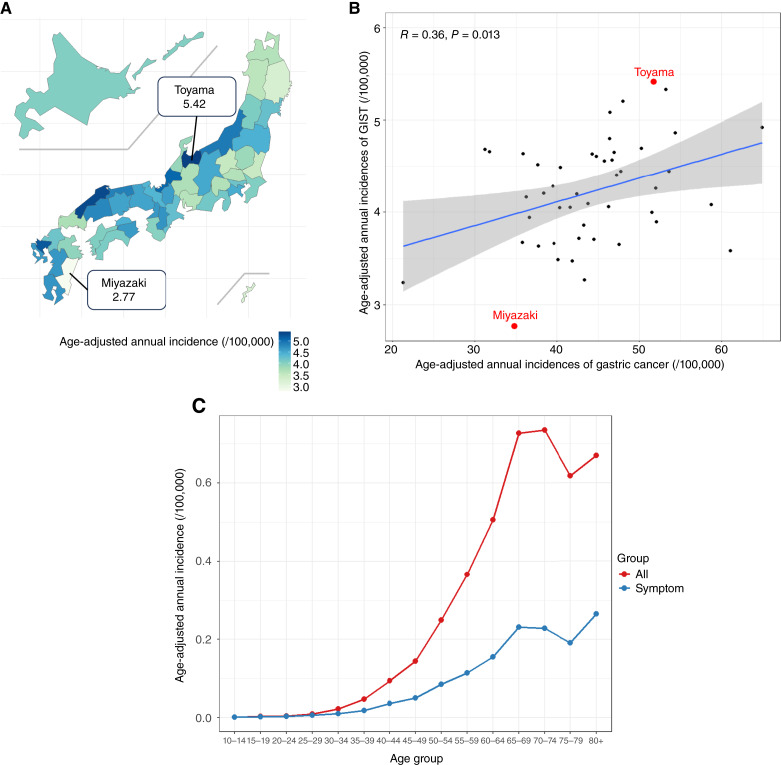
Age-adjusted annual incidence of GIST. **A,** Age-adjusted annual incidence of all cases of newly diagnosed GIST among different prefectures. **B,** Correlation between age-adjusted annual incidence of gastric cancer during 2016 and 2019 and age-adjusted annual incidence of cases of newly diagnosed GIST among different prefectures. **C,** Age-adjusted annual incidence by age groups.

The crude annual incidence of all cases of newly diagnosed GIST during 2016 and 2019 was 4.23 per 100,000 population. The age-adjusted annual incidence of all cases of newly diagnosed GIST during 2016 and 2019 was 4.20 per 100,000 population. The crude annual incidence of GIST newly diagnosed because of symptoms during 2016 and 2019 was 1.41 per 100,000 population. The age-adjusted annual incidence of cases of GIST newly diagnosed because of symptoms during 2016 and 2019 was 1.40 per 100,000 population. There are 47 prefectures in Japan. Among the different prefectures, the age-adjusted annual incidence of all cases of newly diagnosed GIST per 100,000 population ranged from 2.77 (Miyazaki prefecture) to 5.42 (Toyama prefecture) during 2016 and 2019 (Fig. 2A). There was a significant positive correlation between the age-adjusted annual incidence of gastric cancer during 2016 and 2019 and the age-adjusted annual incidence of GIST across prefectures (*R* = 0.36, *P* = 0.013; Fig. 2B). Among the 47 prefectures in Japan, Toyama ranked ninth in annual gastric cancer incidence (51.71 per 100,000), whereas Miyazaki ranked 44th (34.77 per 100,000), representing an approximately 1.49-fold difference. Among the different prefectures, the age-adjusted annual incidence of cases of GIST newly diagnosed because of symptoms per 100,000 population ranged from 1.00 (Yamanashi prefecture) to 2.00 (Shiga prefecture) during 2016 and 2019 (Supplementary Fig. S1). There was a significant positive correlation between the age-adjusted annual incidence of all cases of newly diagnosed GIST and the age-adjusted annual incidence of cases of GIST diagnosed because of symptoms (*R* = 0.48, *P* < 0.001; Supplementary Fig. S2). The 70 to 74 year age group had the highest age-adjusted annual incidence of all cases of newly diagnosed GIST and the ≥80 year age group had the highest age-adjusted annual incidence of cases of GIST newly diagnosed because of symptoms (Fig. 2C).

### OS according to primary organ

**Figure 3:**
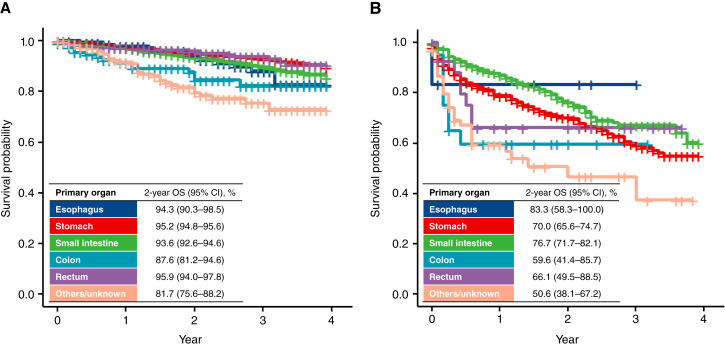
OS by primary organ. **A,** Patients without distant metastasis. **B,** Patients with distant metastasis. Cases in which GIST was detected at autopsy were excluded from analyses.

The median follow-up duration was 702 [95% confidence interval (CI), 692–712] days for all patients. Among patients without distant metastasis, the 2-year OS rates according to the primary organ were as follows: 94.3% for esophageal GIST, 95.2% for gastric GIST, 93.6% for small intestine GIST, 87.6% for colon GIST, 95.9% for rectal GIST, and 81.7% for patients with other/unknown GIST (Fig. 3A). Among patients with distant metastasis, the 2-year OS was 83.3% for patients with esophageal GIST, 70.0% for patients with gastric GIST, 76.7% for patients with small intestine GIST, 59.6% for patients with colon GIST, 66.1% for patients with rectal GIST, and 50.6% for other/unknown GIST (Fig. 3B). OS stratified by primary organ, age group, and sex is presented in Supplementary Figures (without distant metastasis: Supplementary Fig. S3A–S3R; with distant metastasis: Supplementary Fig. S4A–S4R).

### Treatment patterns of patients without distant metastasis between age groups

A total of 18,140 patients were diagnosed with GIST without distant metastasis at the time of the diagnosis. Surgical resection was less frequently performed in the geriatric group (87%) relative to the PAYA (93%) and the adult (93%) groups (*P* < 0.001; Table 2). Moreover, the geriatric group (10%) was less likely to receive chemotherapy than the PAYA (22%) and the adult (16%) groups (*P* < 0.001; Table 2).

**Table 2:** Treatment patterns stratified by age group

Patients without distant metastasis^a^	PAYA (*N* = 359)		Adult (*N* = 11,935)		Geriatric (*N* = 5,846)		*P* value
Surgical resection	​	​	​	​	​	​	​
Performed	332	93%	11,116	93%	5,102	87%	<0.001^b^
Chemotherapy	​	​	​	​	​	​	​
Performed	80	22%	1,920	16%	573	10%	<0.001^b^

Patients with distant metastasis^a^	PAYA (*N* = 20)		Adult (*N* = 652)		Geriatric (*N* = 337)		*P* value
Surgical resection	​	​	​	​	​	​	​
Performed	13	65%	320	49%	138	41%	0.031^b^
Chemotherapy	​	​	​	​	​	​	​
Performed	18	90%	564	87%	205	61%	<0.001^b^

a Cases in which GIST was detected at autopsy were excluded from the analyses.

b Cases with unknown data were excluded from the analyses.

The treatment patterns stratified by both age group and sex were evaluated (Supplementary Fig. S5A–S5F). In the geriatric group, patients aged ≥80 years tended to have lower rates of surgical resection and chemotherapy administration compared with those aged 75 to 79 years. In addition, there was a tendency toward lower rates of chemotherapy administration among younger individuals within the PAYA group.

**Figure 4:**
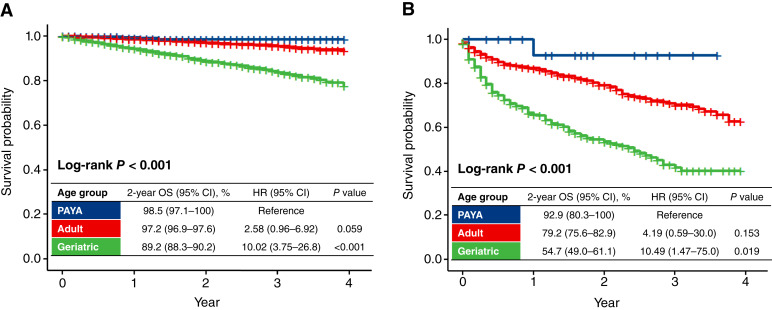
OS by age groups. **A,** Patients without distant metastasis. **B,** Patients with distant metastasis. Cases in which GIST was detected at autopsy were excluded from analyses.

The 2-year OS rates in the PAYA, adult, and geriatric groups were 98.5%, 97.2%, and 89.2%, respectively (log-rank *P* < 0.001; Fig. 4A). The OS of the geriatric group was significantly poorer than that of the PAYA group (HR = 10.02; 95% CI, 3.75–26.8; *P* < 0.001). Among the geriatric group, patients aged ≥80 years had significantly poorer OS than those aged 75 to 79 years, both in the total cohort and in sex-stratified analyses (Supplementary Fig. S6A–S6C).

### Management of patients with distant metastasis according to age group

A total of 1,009 patients were diagnosed with GIST with distant metastasis at diagnosis. Surgical resection was less frequently performed in the geriatric group (41%) relative to the PAYA (65%) and adult (49%) groups (*P* = 0.031; Table 2). Moreover, the geriatric group (61%) was less likely to receive chemotherapy than the PAYA (90%) and adult (87%) groups (*P* < 0.001; Table 2). The treatment patterns stratified by both age group and sex are shown in Supplementary Fig. S7A–S7F. In the geriatric group, a lower frequency of surgical resection and chemotherapy administration was observed among patients aged ≥80 years compared with those aged 75 to 79 years. The 2-year OS rates in the PAYA, adult, and geriatric groups were 92.9%, 79.2%, and 54.7%, respectively (log-rank *P* < 0.001; Fig. 4B). The OS of the geriatric group was significantly poorer than that of the PAYA group (HR = 10.49; 95% CI, 1.47–75.0; *P* = 0.019). In the geriatric population, OS was significantly poorer in patients aged ≥80 years compared with those aged 75 to 79 years, in both the total cohort and analyses stratified by sex (Supplementary Fig S8A–S8C).

## Discussion

To our knowledge, the present study is the first to characterize epidemiologic features of GIST on a national basis (*N* = 21,426) in Japan and to describe several important findings. First, we revealed current demographic indicators (e.g., age-adjusted annual incidence of GIST), differences in incidence by prefecture, and an age-related increase in incidence. Second, we obtained an overview of the epidemiologic characteristics of Japanese patients with GIST. Third, we demonstrated the differences in treatment patterns and prognosis between the geriatric age group and the PAYA and adult age groups in both the nonmetastatic and metastatic settings.

The incidence of GIST has globally been increasing over time for several potential reasons, including penetration of the disease concept and improvements in diagnostic techniques (1). Considering the annual incidence reported in a previous systematic review, the age-adjusted annual incidence of GIST in Japan is one of the highest in the world, at approximately 4 per 100,000 (1). Several underlying reasons might explain this observation. First, the Japanese cancer registry comprehensively collects information on all cancer occurrences regardless of symptoms. Given the lower incidence of GIST diagnosed because of symptoms in this study compared with data from other countries (33% vs. 81%), a higher proportion of Japanese patients might have been diagnosed with small GISTs eligible for active surveillance (1). Second, a nationwide gastric cancer screening program in Japan coupled with the high implementation of gastrectomy may be responsible for the high incidence of gastric GIST in Japan. Microscopic gastric GISTs are frequently found in resected stomach specimens during pathologic examinations (10). Additionally, we observed a considerable variation in the incidence of GIST between prefectures. In Japan, differences in the incidence of various types of cancer (e.g., gastric cancer and liver cancer) at the prefectural level have been reported (11). We identified a positive correlation between the incidence of gastric cancer and that of GIST, suggesting that GIST may be incidentally identified as a result of the diagnostic or therapeutic approach for gastric cancer. In our study, a difference of approximately 1.49 times was observed when comparing the annual incidence of gastric cancer in Toyama prefecture, which has the highest annual incidence of GIST, and Miyazaki prefecture, which has the lowest. The underlying mechanisms explaining this regional variation in the incidence of GIST might be unclarified etiologic factors and non-etiologic factors that require further investigation. The stomach and the small intestine were the major primary sites of GIST in Japan, in line with prior research (1). It has been demonstrated that with advancing age, the proportion of small intestinal GISTs decreases, whereas the proportion of gastric GISTs increases, consistent with a previous study conducted in Taiwan (12). The finding that 5% of patients had distant metastasis at the time of the diagnosis is an important baseline for estimating the number of patients who are eligible for drug therapy in Japan. We found that patients with GIST achieved favorable OS (>80%) at 2 years regardless of the primary site. Even for GIST with distant metastasis, the 2-year OS rate was approximately >50%, possibly because of the availability of drug options. Our study shows that approximately one in three patients with GIST in Japan—which is the world’s most aged society—is in the geriatric population (age ≥75 years). In the context of global aging, it is anticipated that the prevalence of older adults with GIST will increase, and it will become increasingly important to develop treatment strategies that accommodate the unique vulnerabilities of this population (e.g., physical frailty and comorbidities).

Surgical resection is the primary treatment with curative intent for nonmetastatic GIST (13). Adjuvant treatment with imatinib is associated with improved survival of resected GIST with a high risk of recurrence (14). Our results showed that geriatric patients with nonmetastatic GIST underwent surgical resection and chemotherapy less frequently than non-geriatric patients. Although the exact reasons for inactive care in geriatric patients were not clarified, it could be due to physician or patient preferences, considering the risks related to surgery or chemotherapy. A previous study reported that the incidences of perioperative complications and reoperations did not differ between older patients and non-older patients (5). Thus, surgical resection is considered a reasonable curative treatment for properly selected older patients without compromising safety. With regard to imatinib treatment for older patients, the incidence of adverse events is higher than that in non-older patients. Thus, careful management, such as dose modification and fine-tuning of supportive therapy, is required in this population (8). In our analysis, geriatric patients without distant metastasis showed poorer OS than non-geriatric patients. In addition to a low implementation rate of standard treatment, the involvement of deaths from other causes associated with old age might also be considered (15).

Palliative chemotherapy, aimed at prolonging survival and disease control, is the standard treatment for patients who have GIST with distant metastasis (13). As a first-line treatment, imatinib confers a long-term survival benefit of approximately 4 years in these patients (16, 17). Our study showed that geriatric patients underwent palliative chemotherapy less frequently than non-geriatric patients, which is in line with a previous study (5). As previously mentioned, imatinib is associated with increased incidences of severe adverse events and dose reduction in older patients; thus, caution is required in treatment management (8). Nonetheless, previous studies have suggested the non-inferiority of imatinib efficacy in terms of progression-free survival between older and non-older patients (5, 8). Geriatric patients receiving chemotherapy may have age-specific problems such as reduced organ function, cognitive decline, and comorbidities. Accordingly, in this age group, geriatric assessment may be useful to identify vulnerabilities that might be overlooked in a standard clinical assessment (18).

Cytoreductive surgery may play a role in removing drug-resistant tumor cells or delaying their emergence, thereby prolonging survival for patients with GIST and distant metastasis. Clinical guidelines suggest considering cytoreductive surgery for patients with metastatic disease, such as in cases with long-term disease stability after drug therapy or at the time of focal disease progression (2, 4). Our study showed a trend toward not performing surgical resection for metastatic GIST in geriatric patients, consistent with a previous report using the Surveillance, Epidemiology, and End Results database (19). However, from the available data in the NCR, it is unclear why surgery was performed on such a large proportion of patients with metastatic disease.

The present study had several limitations. The NCR does not capture some clinicopathologic information specific to GIST, including tumor–node–metastasis stage, risk classification of resected GIST, genetic mutations of *KIT* and *PDGFRA*, specific drug names and doses of chemotherapy, and the efficacy and safety of each treatment. Discussions related to the use of imatinib as adjuvant or first-line treatment are based on the assumption that it is widely used as standard treatment in clinical practice in Japan rather than on specific data from the NCR. Additionally, clinical data such as performance status, comorbidities, and geriatric assessment and outcomes, including relapse-free survival and progression-free survival, are not available. Therefore, no adjustment for clinicopathologic factors was performed in this study. Epidemiologic changes over the years and matured data on OS are not available at this moment. Another limitation of our study is the lack of cause-of-death information and age-matched general population survival data, which does not allow us to assess GIST-specific survival or compare the survival of nonmetastatic patients with that of the general population. Future studies incorporating linked datasets, such as national mortality statistics, would be valuable for evaluating disease-specific outcomes.

In conclusion, the annual incidence of GIST in Japan is among the highest in the world, with geriatric patients accounting for approximately 30% of all cases. Geriatric patients experienced substantial undertreatment relative to non-geriatric patients. Our findings support a rationale for designing clinical studies to understand clinical practices and develop guidelines to optimize the management of the geriatric population.

### Ethics statement

Following the procedures stipulated in the Cancer Registry Act, this study was reviewed by the Data Utilization Committee of the NCR Office. In accordance with the ethical guidelines for human subject research in Japan, this study was exempted from ethical review by the Institutional Review Board (20). The publication is based on information provided in accordance with the law and includes independently created and processed materials.

## Supplementary Material

Supplementary Fig. S1 Supplementary Fig. S1. Age-adjusted annual incidence of cases of newly diagnosed GIST by symptom among different prefectures GIST, gastrointestinal stromal tumor.

Supplementary Fig. S2 Supplementary Fig. S2. Correlation between age-adjusted annual incidence of all cases of newly diagnosed GIST and age-adjusted annual incidence of cases of newly diagnosed GIST by symptom among different prefectures GIST, gastrointestinal stromal tumor.

Supplementary Fig. S3 Supplementary Fig. S3. Overall survival stratified by age group, sex, and primary tumor site in patients without distant metastasis.

Supplementary Fig. S4 Supplementary Fig. S4. Overall survival stratified by age group, sex, and primary tumor site in patients with distant metastasis.

Supplementary Fig. S5 Supplementary Fig. S5. Treatment patterns stratified by age group and sex in patients without distant metastasis.

Supplementary Fig. S6 Supplementary Fig. S6. Overall survival among geriatric patients with stratified without distant metastasis stratified by age subgroup.

Supplementary Fig. S7 Supplementary Fig. S7. Treatment patterns stratified by age group and sex in patients with distant metastasis.

Supplementary Fig. S8 Supplementary Fig. S8. Overall survival among geriatric patients with stratified with distant metastasis stratified by age subgroup.
