# Supplementary material for: Epidemiologic Features and Age-Related Differences in Management among Patients with Gastrointestinal Stromal Tumors in Japan: A National Cancer Registry Study
Source: Cancer Res Commun. 2025 Jul 29;5(7):1235–42. doi: 10.1158/2767-9764.CRC-25-0074 (PMC12304871; doi:10.1158/2767-9764.CRC-25-0074)
Supplement: Supplementary Fig. S1 — Age-adjusted annual incidence of cases of newly diagnosed GIST by symptom among different prefectures GIST, gastrointestinal stromal tumor. [file crc-25-0074_supplementary_fig.s1_suppsf1.docx]

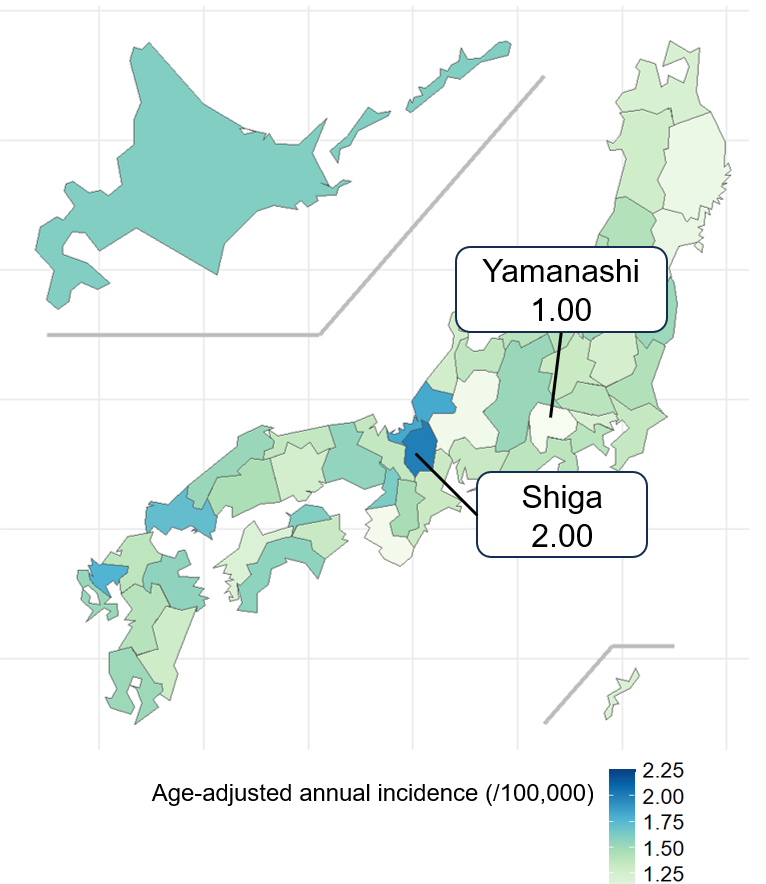


# **Supplementary Fig. S1**

Age-adjusted annual incidence of cases of newly diagnosed GIST by symptom among different prefectures

GIST, gastrointestinal stromal tumor.
