# Supplementary material for: Epidemiologic Features and Age-Related Differences in Management among Patients with Gastrointestinal Stromal Tumors in Japan: A National Cancer Registry Study
Source: Cancer Res Commun. 2025 Jul 29;5(7):1235–42. doi: 10.1158/2767-9764.CRC-25-0074 (PMC12304871; doi:10.1158/2767-9764.CRC-25-0074)
Supplement: Supplementary Fig. S3 — Overall survival stratified by age group, sex, and primary tumor site in patients without distant metastasis. [file crc-25-0074_supplementary_fig.s3_suppsf3.docx]

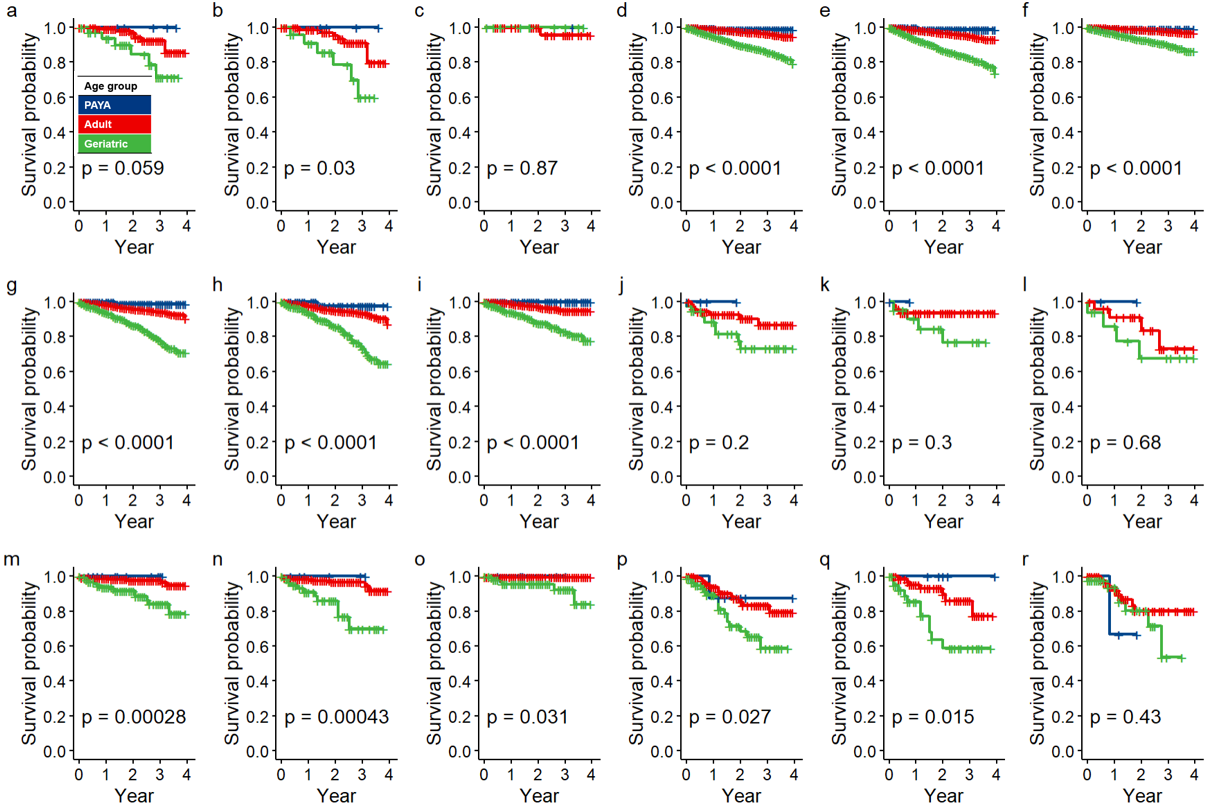


# Supplementary Fig. S3

Overall survival stratified by age group, sex, and primary tumor site in patients without distant metastasis

**a** Esophageal GIST (total patients). **b** Esophageal GIST (male patients). **c** Esophageal GIST (female patients). **d** Gastric GIST (total patients). **e** Gastric GIST (male patients). **f** Gastric GIST (female patients). **g** Small intestinal GIST (total patients). **h** Small intestinal GIST (male patients). **i** Small intestinal GIST (female patients). **j** Colon GIST (total patients). **k** Colon GIST (male patients). **l** Colon GIST (female patients). **m** Rectal GIST (total patients). **n** Rectal GIST (male patients). **o** Rectal GIST (female patients). **p** Others/unknown GIST (total patients). **q** Others/unknown (male patients). **r** Others/unknown (female patients). OS cureved are color-coded by age group: blue for PAYA patients, red for adult patients, and green for geriatric patients. Cases in which GIST was detected at autopsy were excluded from analyses. GIST, gastrointestinal stromal tumor; OS, overall survival; PAYA, pediatric and adolescent young adult.
