# Supplementary material for: Epidemiologic Features and Age-Related Differences in Management among Patients with Gastrointestinal Stromal Tumors in Japan: A National Cancer Registry Study
Source: Cancer Res Commun. 2025 Jul 29;5(7):1235–42. doi: 10.1158/2767-9764.CRC-25-0074 (PMC12304871; doi:10.1158/2767-9764.CRC-25-0074)
Supplement: Supplementary Fig. S7 — Treatment patterns stratified by age group and sex in patients with distant metastasis. [file crc-25-0074_supplementary_fig.s7_suppsf7.docx]

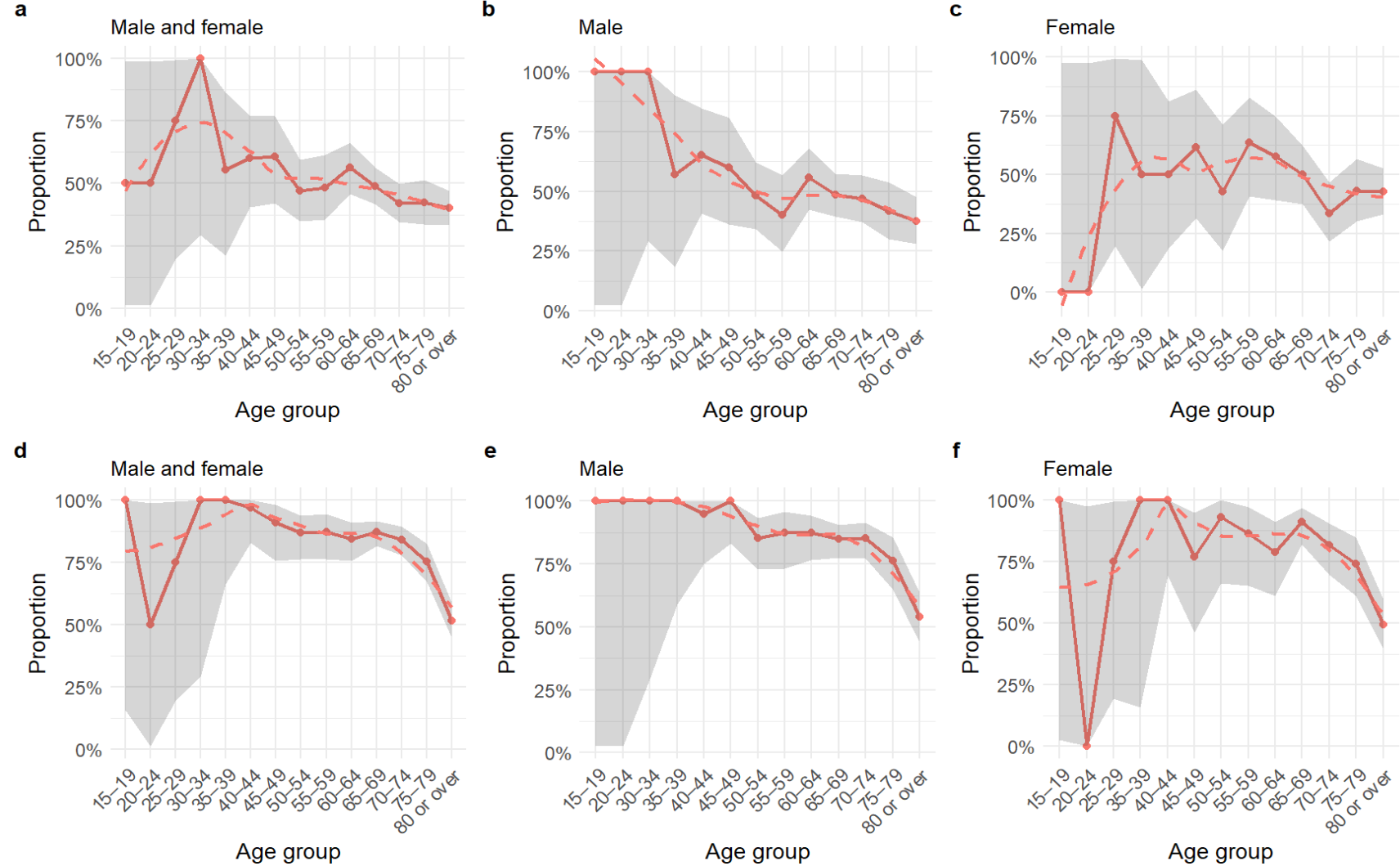


# Supplementary Fig. S7

Treatment patterns stratified by age group and sex in patients with distant metastasis

**a** Proportion of patients receiving surgical resection (total patients). **b** Proportion of patients receiving surgical resection (male patients). **c** Proportion of patients receiving surgical resection (female patients). **d** Proportion of patients receiving chemotherapy (total patients). **e** Proportion of patients receiving chemotherapy (male patients). **f** Proportion of patients receiving chemotherapy (female patients). The solid line represents the observed proportion of patients receiving the specified treatment across age groups. The shaded area indicates the 95% confidence interval calculated using the binomial test for each age group. A dashed line shows the fitted trend using a locally estimated scatterplot smoothing method.
