# Supplementary material for: Epidemiologic Features and Age-Related Differences in Management among Patients with Gastrointestinal Stromal Tumors in Japan: A National Cancer Registry Study
Source: Cancer Res Commun. 2025 Jul 29;5(7):1235–42. doi: 10.1158/2767-9764.CRC-25-0074 (PMC12304871; doi:10.1158/2767-9764.CRC-25-0074)
Supplement: Supplementary Fig. S8 — Overall survival among geriatric patients with stratified with distant metastasis stratified by age subgroup. [file crc-25-0074_supplementary_fig.s8_suppsf8.docx]

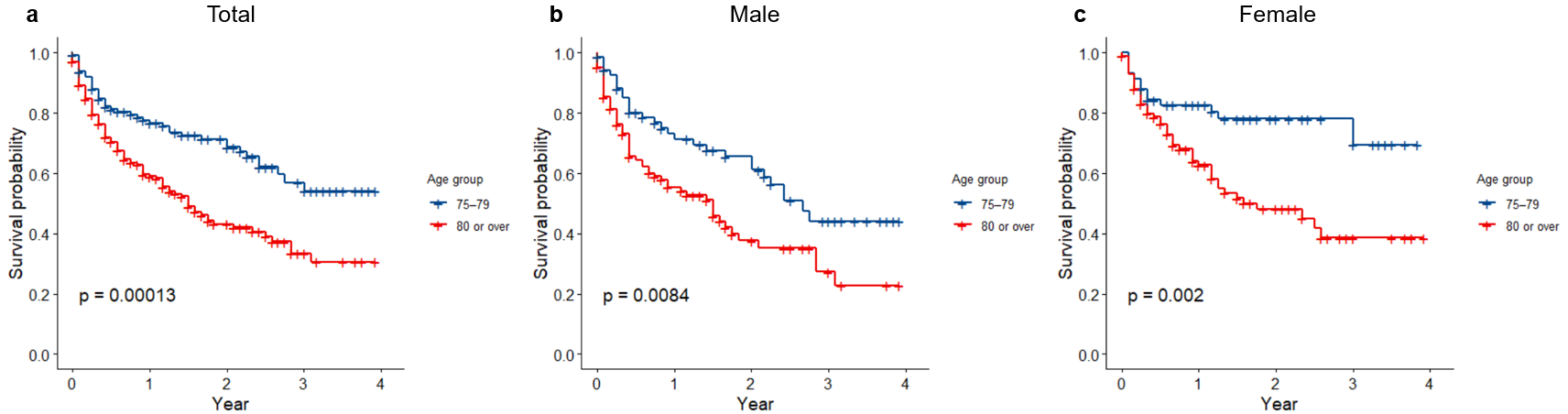


# Supplementary Fig. S8

Overall survival among geriatric patients with stratified with distant metastasis stratified by age subgroup.

**a** Total patients. **b** Male patients. **c** Female patients.
